# The impact of circulating IGF-1 and IGFBP-2 on cardiovascular prognosis in patients with acute coronary syndrome

**DOI:** 10.3389/fcvm.2023.1126093

**Published:** 2023-03-10

**Authors:** Wei Wang, Kang Yu, Shou-Yong Zhao, De-Gang Mo, Jia-Hui Liu, Li-Jinn Han, Tai Li, Heng-Chen Yao

**Affiliations:** ^1^ Department of Cardiology, Liaocheng People's Hospital, Shandong University, Jinan, China; ^2^ Department of Cardiology, Liaocheng People's Hospital Affiliated to Shandong First Medical University, Liaocheng, China; ^3^ Department of Laboratory Medicine, Liaocheng People's Hospital Affiliated to Shandong First Medical University, Liaocheng, China; ^4^ Department of Nursing, Liaocheng Vocational & Technical College, Liaocheng, China

**Keywords:** acute coronary syndrome, insulin-like growth factor-1, insulin-like growth factor binding protein 2, cardiovascular prognosis, IGF-1, IGFBP-2

## Abstract

**Background:**

While insulin-like growth factor 1 (IGF-1) exerts a cardioprotective effect in the setting of atherosclerosis, insulin-like growth factor binding protein 2 (IGFBP-2) is involved in metabolic syndrome. Although IGF-1 and IGFBP-2 are known to be predictors for mortality in patients with heart failure, their use in clinic as prognostic biomarkers for acute coronary syndrome (ACS) requires investigation. We evaluated the relationship between IGF-1 and IGFBP-2 levels at admission and the risk of major adverse cardiovascular events (MACEs) in patients with ACS.

**Methods:**

A total of 277 ACS patients and 42 healthy controls were included in this prospective cohort study. Plasma samples were obtained and analyzed at admission. Patients were followed for MACEs after hospitalization.

**Results:**

Among patients who suffered acute myocardial infarction, plasma levels of IGF-1 and IGFBP-2 were lower and higher, respectively, as compared to healthy controls (both *p* < 0.05). The mean follow-up period was 5.22 (1.0–6.0) months and MACEs incidence was 22.4% (62 of 277 patients). Kaplan–Meier survival analysis revealed that patients with low IGFBP-2 levels had a greater event-free survival rate than patients with high IGFBP-2 levels (*p* < 0.001). Multivariate Cox proportional hazards analysis revealed IGFBP-2, but not IGF-1, to be a positive predictor of MACEs (hazard ratio 2.412, 95% CI 1.360–4.277; *p* = 0.003).

**Conclusion:**

Our findings suggest that high IGFBP-2 levels are associated with the development of MACEs following ACS. Moreover, IGFBP-2 is likely an independent predictive marker of clinical outcomes in ACS.

## Introduction

Acute coronary syndrome (ACS) is characterized by a sudden decrease in blood flow to the heart. Worldwide, more than seven million people are annually diagnosed with ACS; approximately 5% of this patient population was reported to die prior to hospital discharge (1, 2). Although scientific advances have greatly facilitated implementation of effective secondary cardiovascular prevention strategies, previously unrecognized mediators of cardiovascular disease (CVD) continue to be discovered.

Insulin-like growth factors (IGFs) are conserved peptide hormones structurally similar to insulin that are expressed universally in multiple tissues (3). Interestingly, IGF-1 is not only found in the circulation but also in arteries, with studies having reported IGF-1 to exert cardioprotective effects in the setting of atherosclerosis (4, 5). Preclinical model studies reported that administration of IGF-1 suppresses cardiac fibrosis induced by angiotensin II (6), and treatment of sheep fetuses with IGF-1 was reported to stimulate growth of the coronary vasculature and myocardium (7). Furthermore, bone marrow mesenchymal stem cells overexpressing IGF-1 were reported to better resist apoptosis in myocardial infarction (8). In clinical practice, serum IGF-1 levels are decreased in heart failure (HF) patients (9) and serve as a predictor of cardiovascular mortality in this condition (10).

All six members of the IGF-binding protein (IGFBP) family regulate IGF bioavailability (11, 12). As the second most abundant protein of this family (13), IGFBP-2 plays critical roles in several pathological processes including carcinogenesis (14), pulmonary arterial hypertension (PAH) (15), obesity and insulin resistance (13). In addition to strongly predicting mortality in HF patients (16), IGFBP-2 has recently emerged as a novel candidate biomarker for cardiovascular risk assessment in patients with aortic stenosis who undergo transcatheter aortic valve implantation (17) and elderly men (18).

To date, relevant research has been limited to animal experiments, preclinical analyses or patients with HF, PAH or aortic stenosis. As such, the prognostic influences of circulating IGF-1 and IGFBP-2 in ACS patients remain unclear. Here, we evaluated the relationship of circulating IGF-1 and IGFBP-2 with major adverse cardiovascular events (MACEs) in ACS patients who underwent coronary angiography (CAG).

## Methods

### Study design and population

This study was a prospective cohort study conducted according to regulations set forth by the Declaration of Helsinki. This study was approved by the Ethics Committee of Liaocheng People's Hospital and all subjects enrolled provided informed consent. From June 1 2021 to October 1 2021, 304 ACS patients who underwent CAG and 42 site-matched controls free of clinical heart disease were initially enrolled in this study. All ACS patients enrolled met relevant diagnostic criteria for either ST-elevation myocardial infarction (STEMI) or non-ST-elevation ACS (19, 20). Patients with severe liver disease or renal failure, neoplasms of any kind or infectious or inflammatory conditions were excluded from this study. Patients lost to follow-up were also excluded from analyses. The treatment therapy, including intensive treatment with medicine, percutaneous coronary intervention (PCI) or coronary artery bypass grafting (CABG) was decided by two experienced cardiologists according to the results of CAG and international standards and guidelines (21).

### Laboratory measurement

Venous blood samples were draw from study subjects for evaluation prior to administration of any medications. Blood samples were collected using tubes containing ethylenediaminetetraacetic acid and centrifuged at 3,000 rpm for 15 min immediately after collection. Samples were aliquoted and stored at −80°C until use. Plasma levels of IGF-1 and IGFBP-2 were assayed using the enzyme linked immunosorbent assays (ELISA) IGF-1 (DG100B) and IGFBP-2 (DGB 200) (both R&D Systems, USA), according to manufacturer's protocol. No significant cross-reactivity or interference of IGFBP/IGF-1 with IGFBP-2 was found in the immunoassay according to product description. ELISA IGFBP-2 kit did not measure IGFBP in complex with IGF-1.

### Definitions

Participants with one major coronary artery ≥50% stenosis were considered as single-vessel disease, whereas multi-vessel disease (MVD) was defined in cases of stenoses ≥50% in 2 or 3 major epicardial coronary arteries. Stenosis of the left main coronary artery ≥50% was regarded as the left main disease (22). Incomplete revascularization (IR) was defined as one or more vessels stenosis ≥50% being left untreated after revascularization (23).

### Follow-up and outcomes

The entire cohort was followed up for 6 months starting from date of hospitalization. Data were systematically obtained *via* phone interviews and review of medical records. A total of 27 ACS patients were lost of follow-up and excluded from this study. A total of 277 ACS patients who completed follow-up were finally evaluated and included 93 unstable angina (UA), 89 non-ST-elevation myocardial infarction (NSTEMI) and 95 STEMI patients. Cardiovascular death, angina, new-onset HF, recurrent myocardial infarction (MI) or any revascularization were all defined as MACEs.

### Statistical analyses

The Shapiro–Wilk test was used to determine whether continuous data were normally distributed. Normally distributed continuous variables were expressed as mean ± standard deviation; data not normally distributed were expressed as median (interquartile range). Comparisons of continuous variables between two groups were performed using the Mann–Whitney U test or t-test. Comparisons of continuous variables among four groups were performed using one-way analysis of variance or the Kruskal–Wallis H test. The pairwise test for multiple comparisons was used to analyze intergroup differences for IGF-1 and IGFBP-2 levels after Kruskal–Wallis H analysis. Categorical variables were presented as frequency and percentage and compared using the chi-squared test. Spearman correlation analysis was performed to analyze the correlation of plasma IGF-1 and IGFBP-2 levels.

A receiver operator characteristics (ROC) curve was generated and the area under the curve (AUC) was calculated, and Z test were used to compare AUC values. Optimal IGF-1 and IGFBP-2 cutoff points for MACEs prediction were determined based on maximal Youden's index. Kaplan–Meier survival curves were constructed to analyze the short-term event-free survival (EFS) rate and comparisons were performed using the log-rank test. Cox proportional hazards regression analysis was performed to determine independent factors predictive for MACEs; confounders with unadjusted *p*-values <0.05 in univariate analysis were included in a multivariate regression model. A two-tailed *p*-value of <0.05 was considered as statistically significant. Statistical analyses were performed using SPSS 23.0 (IBM, USA).

## Results

### Clinical characteristics

Median values (interquartile ranges) of IGF-1 and IGFBP-2 concentrations in healthy controls (*n* = 42) were 170.39 (106.14) ng/ml and 199.1 (255.01) ng/ml, respectively. While IGF-1 levels in healthy controls were higher than in patients who suffered NSTEMI or STEMI (*p* < 0.05), IGFBP-2 levels showed the opposite pattern. Although IGFBP-2 levels in STEMI patients were higher than those in UA patients (*p* < 0.05), no significant difference between healthy controls and UA patients was found (*p* > 0.05; Table 1).

**Table 1:** Plasma levels of IGF-1 and IGFBP-2 in studied population.

Variables	HC (*n* = 42)	UA (*n* = 93)	NSTEMI (*n* = 89)	STEMI (*n* = 95)
IGF-1 (ng/ml)	170.39 (106.14)	138.89 (86.53)	117.53 (76.31)^a^	117.95 (96.6)^a^
IGFBP-2 (ng/ml)	199.1 (255.01)	258.74 (245.03)	318.55 (394.59)^a^	364.75 (400.79)^a,b^

^a^ *p* < 0.05 when compared NSTEMI or STEMI with HC.

^b^ *p* < 0.05 when compared STEMI with UA.

IGF-1, Insulin like growth factor 1; IGFBP-2, Insulin like growth factor binding protein 2; HC, Healthy control; UA, Unstable angina; NSTEMI, Non-ST-segment elevation myocardial infarction; STEMI, ST-segment elevation myocardial infarction.

In a total of 277 ACS patients, 195 (70.4%) presented with MVD, 37 (13.4%) presented with left main vessel disease, and the number of patients with IR treatment was 32 (11.6%). 89 (32.1%) patients were treated with intensive medication, and 188 (67.9%) patients were treated with PCI or CABG. Patients were divided into two groups based on median IGF-1 concentrations: a high IGF-1 group (IGF-1 levels ≥126.92 ng/ml; *n* = 139) and a low IGF-1 group (IGF-1 levels <126.92 ng/ml; *n* = 138). High IGF-1 group patients were found to have a higher ejection fraction (EF), a lower rate of acute myocardial infarction (AMI) and a higher rate of multi-vessel lesion as compared to those in the low IGF-1 group. No other statistically significant differences in demographic characteristics or laboratory evaluations were noted between the two groups (Table 2).

**Table 2:** Basic characteristics of studied patients according to plasma IGF-1 levels.

Characteristics	IGF-1 ≧ 126.92 ng/ml	IGF-1 < 126.92 ng/ml	*p* value
	*n* = 139	*n* = 138	
Age (years)	59 ± 12	62 ± 12	0.491
Male, *n* (%)	111 (79.9)	102 (73.9)	0.241
Smoking, *n* (%)	77 (55.4)	73 (52.9)	0.677
Hypertension, *n* (%)	85 (61.2)	74 (53.6)	0.205
DM, *n* (%)	37 (26.6)	38 (27.5)	0.864
Heart rate (bpm)	72 (17)	72 (17)	0.584
BMI (kg/m^2^)	25.78 ± 3.75	25.25 (4.3)	0.137
Hemoglobin (g/L)	137 ± 16	134 ± 16	0.786
WBC count (×10^9^/L)	7.5 (3.6)	7.7 (3.7)	0.671
D-dimer (mg/L)	0.35 (0.45)	0.35 (0.37)	0.912
Creatinine (umol/L)	67.3 (19.5)	69.6 (18.9)	0.576
TG (mmol/L)	1.35 (1.06)	1.28 (0.80)	0.115
LDL (mmol/L)	2.62 ± 0.79	2.63 (0.94)	0.539
TC (mmol/L)	4.27 ± 1.08	4.29 (1.33)	0.761
CRP (mg/L)	3.55 (6.02)	4.26 (7.48)	0.227
EF (%)	58 (14)	54 (14)	0.025
LVEDD (mm)	45 (5)	46 (6)	0.198
Diagnosis, *n* (%)			
AMI	83 (59.7)	101 (73.2)	0.018
UA	56 (40.1)	37 (26.8)	0.018
Angiography, *n* (%)			
One-vessel lesion	33 (23.7)	49 (35.5)	0.032
Multi-vessel lesion	106 (76.3)	89 (64.5)	0.032
Left main vessel lesion	22 (15.8)	15 (10.8)	0.225
IR	18 (12.9)	14 (10.1)	0.465
Treatment strategies, *n* (%)			
Intensive medication	39 (28.1)	50 (36.2)	0.145
PCI/CABG	100 (71.9)	88 (63.8)	0.145
IGF-1 (ng/ml)	173.6 (78.6)	88.9 (37.3)	<0.001
IGFBP-2 (ng/ml)	259.3 (317.9)	326.53 (394.5)	0.112

IGF-1, Insulin like growth factor 1; DM, Diabetes mellitus; BMI, Body mass index; WBC, White blood cell; TG, Triglyceride; LDL, Low-density lipoprotein; TC, Total cholesterol; CRP, C-reactive protein; EF, Ejection fraction; LVEDD, Left ventricular end-diastolic dimension; AMI, Acute myocardial infarction; UA, Unstable angina; IR, Incomplete revascularization; PCI, Percutaneous coronary intervention; CABG, Coronary artery bypass grafting; IGFBP-2, Insulin like growth factor binding protein 2.

Patients were divided into two groups based on median IGFBP-2 concentrations: a high IGFBP-2 group (IGFBP-2 ≥ 308.3 ng/ml; *n* = 139) and a low IGFBP-2 group (IGFBP-2 < 308.3 ng/ml; *n* = 138). As shown in Table 3, high IGFBP-2 group patients were older, had lower levels of hemoglobin, triglycerides and EF, higher D-dimer levels, and lower body mass indices (BMI). Moreover, high IGFBP-2 group patients were found to have a higher rate of AMI as compared to low IGFBP-2 group patients.

**Table 3:** Basic characteristics of studied patients according to plasma IGFBP-2 levels.

Characteristics	IGFBP-2 ≧ 308.3 ng/ml	IGFBP-2 < 308.3 ng/ml	*p* value
	*n* = 139	*n* = 138	
Age (years)	62 ± 12	58 ± 12	0.006
Male, *n* (%)	108 (77.7)	105 (76.1)	0.750
Smoking, *n* (%)	81 (58.3)	69 (50.0)	0.167
Hypertension, *n* (%)	79 (56.8)	80 (58.0)	0.848
DM, *n* (%)	38 (27.3)	37 (26.8)	0.921
Heart rate (bpm)	74 (18)	70 (16)	0.552
BMI (kg/m^2^)	25.1 ± 3.46	25.9 ± 3.49	0.049
Hemoglobin (g/L)	134 ± 16	138 ± 17	0.034
WBC count (×10^9^/L)	7.59 (3.59)	7.66 (3.45)	0.561
D-dimer (mg/L)	0.4 (0.57)	0.33 (0.34)	0.014
Creatinine (umol/L)	67 (19)	68 (20)	0.143
TG (mmol/L)	1.24 (0.73)	1.45 (0.96)	0.015
LDL (mmol/L)	2.63 ± 0.74	2.67 ± 0.81	0.682
TC (mmol/L)	4.24 ± 1.01	4.26 ± 1.09	0.871
CRP (mg/L)	3.88 (7.41)	3.84 (6.46)	0.421
EF (%)	54 (15)	59 (14)	0.017
LVEDD (mm)	46 (7)	45 (5)	0.174
Diagnosis, *n* (%)			
AMI	103 (74.1)	81 (58.7)	0.007
UA	36 (25.9)	57 (41.3)	0.007
Angiography, *n* (%)			
One-vessel lesion	40 (28.9)	42 (30.4)	0.762
Multi-vessel lesion	99 (71.1)	96 (69.6)	0.762
Left main vessel lesion	17 (12.2)	20 (14.4)	0.580
IR	18 (12.9)	14 (10.1)	0.465
Treatment strategies, *n* (%)			
Intensive medication	38 (27.3)	51 (37)	0.087
PCI/CABG	101 (72.7)	87 (63)	0.087
IGF-1 (ng/ml)	121.9 (82.4)	129.0 (88.4)	0.328
IGFBP-2 (ng/ml)	518.25 (312.63)	187.89 (123.35)	<0.001

IGFBP-2, Insulin like growth factor binding protein 2; DM, Diabetes mellitus; BMI, Body mass index; WBC, White blood cell; TG, Triglyceride; LDL, Low-density lipoprotein; TC, Total cholesterol; CRP, C-reactive protein; EF, Ejection fraction; LVEDD, Left ventricular end-diastolic dimension; AMI, Acute myocardial infarction; UA, Unstable angina; IR, Incomplete revascularization; PCI, Percutaneous coronary intervention; CABG, Coronary artery bypass grafting; IGF-1, Insulin like growth factor 1.

**Figure 1:**
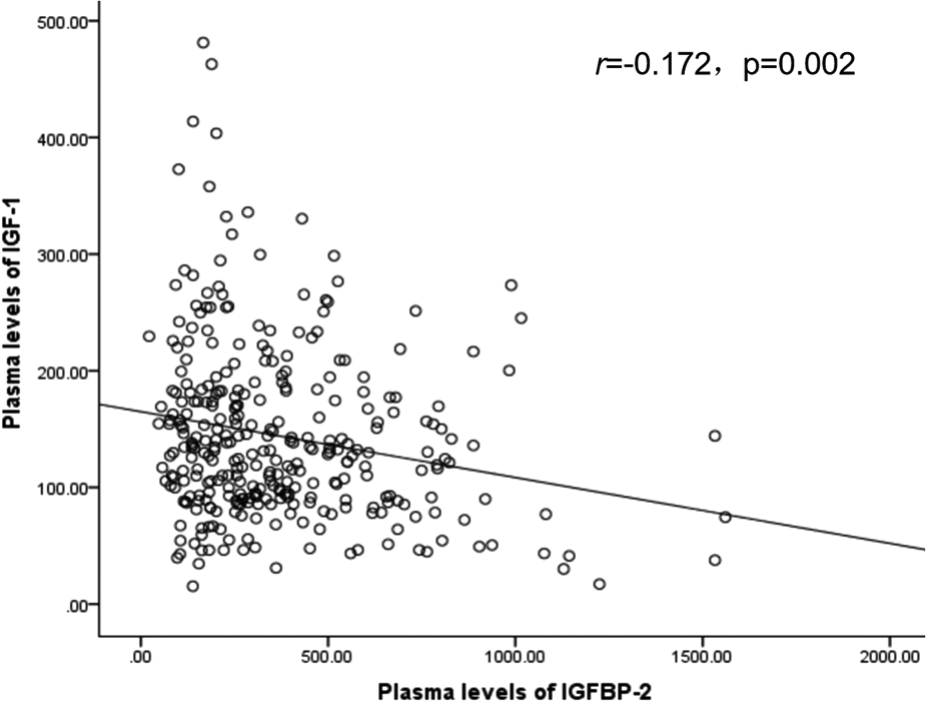
Correlation graph of IGF-1 and IGFBP-2. IGF-1: Insulin like growth factor 1; IGFBP-2: Insulin like growth factor binding protein 2.

The correlation of plasma IGF-1 and IGFBP-2 levels was evaluated by Spearman correlation analysis. A significant negative correlation was found among IGF-1 and IGFBP-2 (*r* = −0.172, *p* = 0.002; Figure 1).

### Clinical outcomes of adverse cardiovascular events

The mean follow-up period was 5.22 (1.0–6.0) months. The incidence of MACEs was 22.4% (62 of 277 patients) and included instances of cardiovascular death (*n* = 8), angina (*n* = 21), HF (*n* = 13) and reinfarction or revascularization (*n* = 20). No patient died of non-cardiovascular causes. No statistical differences in MACEs rates between high and low IGF-1 group patients were found (*p* = 0.141). Total MACEs incidence was higher among high IGFBP-2 group patients as compared to low IGFBP-2 group patients (*p* < 0.001). Furthermore, incidences of angina (*p* = 0.003) and HF (*p* = 0.011) were higher among high IGFBP-2 group patients as compared to low IGFBP-2 group patients (Table 4).

**Table 4:** Major adverse cardiac events according to plasma IGF-1 and IGFBP-2 levels.

Complications	Plasma IGF-1 levels			Plasma IGFBP-2 levels		
	Low (*n* = 138)	High (*n* = 139)	*p* value	Low (*n* = 138)	High (*n* = 139)	*p* value
Death, *n* (%)	5 (3.6)	3 (2.2)	0.467	3 (2.2)	5 (3.6)	0.728
Angina, *n* (%)	11 (8.0)	10 (7.2)	0.807	4 (2.9)	17 (12.2)	0.003
Heart failure, *n* (%)	9 (6.5)	4 (2.9)	0.152	2 (1.4)	11 (7.9)	0.011
Reinfarction or Revascularization, *n* (%)	11 (6.0)	9 (6.5)	0.630	8 (5.8)	12 (8.6)	0.362
Total MACEs, *n* (%)	36 (25.9)	26 (18.8)	0.141	17 (12.3)	45 (32.4)	<0.001

IGF-1, Insulin like growth factor 1; IGFBP-2, Insulin like growth factor binding protein 2; MACEs, Major adverse cardiac events.

### Kaplan-Meier survival curves of circulating IGF-1 and IGFBP-2 in ACS patients during follow-up

**Figure 2:**
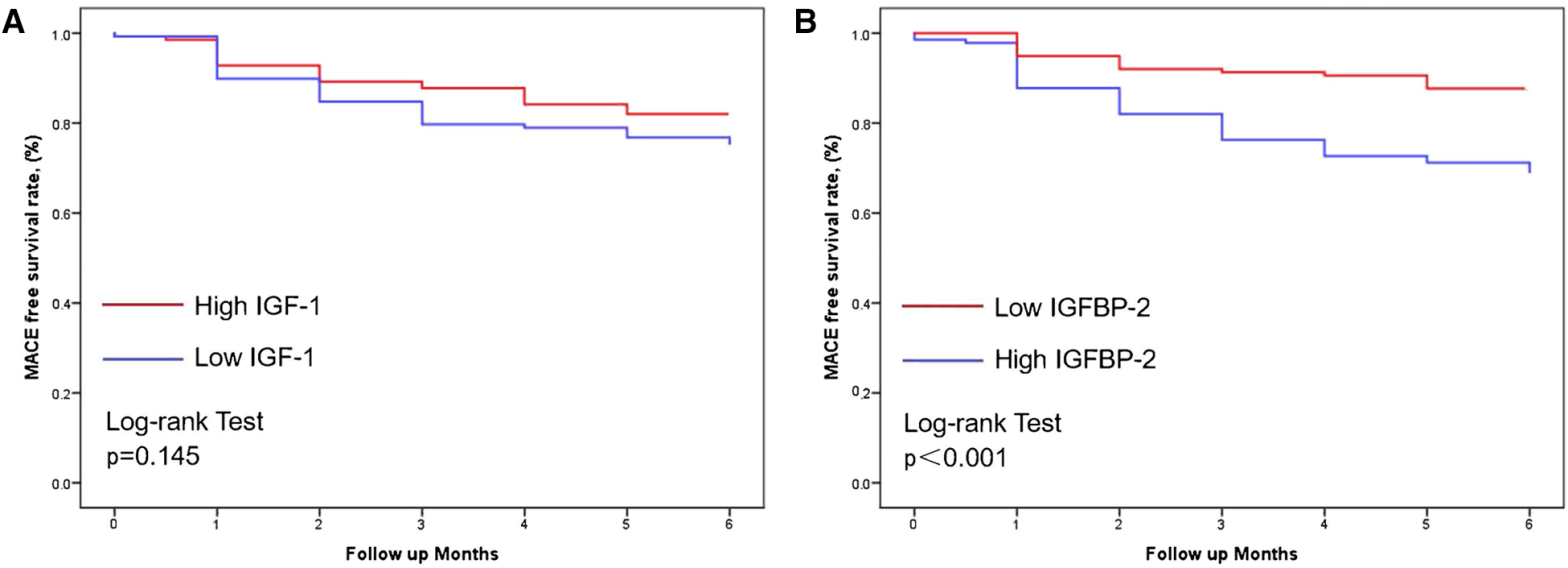
Kaplan-Meier curves in patients with ACS with individual levels of IGF-1 (**A**) and IGFBP-2 (**B**) during follow-up (*p* = 0.145 and <0.001, respectively). MACEs, Major adverse cardiovascular events; ACS, Acute coronary syndrome; IGF-1, Insulin like growth factor 1; IGFBP-2, Insulin like growth factor binding protein 2.

Kaplan-Meier survival analysis revealed no statistically significant differences in event-free survival (EFS) between high and low IGF-1 group patients (*p* = 0.145; Figure 2A). Low IGFBP-2 group patients were found to have had a higher EFS as compared to high IGFBP-2 group patients (*p* < 0.001; Figure 2B). As such, patients with low levels of IGFBP-2 were found to have had a more favorable prognosis as compared to those with high levels of IGFBP-2.

### Independent predictors for MACEs in ACS patients

As shown in Table 5, we analyzed potential confounders for association with short-term MACEs using univariate analysis. Confounders with *p*-values of <0.05 in univariate analysis were included in multivariate Cox regression analysis. After correcting for age, diagnosis of AMI, creatinine level, EF, intensive medication therapy and left main vessel lesion, high IGFBP-2 level was confirmed to have positively predicted value for MACEs [adjusted hazard ratio: 2.412, 95% confidential interval (CI) 1.360–4.277; *p* = 0.003]. Furthermore, EF and left main vessel lesion were found to be independent predictors for MACEs, while IGF-1 level was not.

**Table 5:** Cox proportional hazard analysis for predictors of MACEs.

Variables	HR	95% CI	*p* value	Adjusted HR	95% CI	*p* value
Male	1.295	0.741–2.264	0.363			
Age	1.037	1.014–1.061	0.001	1.016	0.993–1.040	0.175
Smoking status	1.044	0.634–1.720	0.865			
Hypertension	1.062	0.643–1.874	0.816			
DM	1.563	0.929–2.629	0.093			
Diagnosis of AMI	3.319	1.637–6.731	0.001	1.760	0.787–3.936	0.169
Heart rate	1.012	0.996–1.029	0.145			
BMI	0.971	0.957–0.986	0.052			
WBC count	1.031	0.955–1.113	0.440			
Creatinine	1.012	1.005–1.019	0.001	1.006	0.999–1.014	0.110
TG	0.790	0.578–1.079	0.138			
LDL	0.906	0.658–1.247	0.543			
TC	0.869	0.684–1.105	0.252			
CRP	1.010	1.000–1.021	0.054			
EF	0.935	0.911–0.960	<0.001	0.956	0.927–0.985	0.004
Intensive medication	1.762	1.066–2.911	0.027	0.609	0.356–1.043	0.071
Multi-vessel lesion	1.243	0.704–2.196	0.454			
Left main Vessel lesion	2.134	1.193–3.816	0.011	1.868	1.014–3.440	0.045
IR	1.525	0.752–3.091	0.242			
High IGF-1	0.694	0.419–1.150	0.157			
High IGFBP-2	2.894	1.656–5.057	<0.001	2.412	1.360–4.277	0.003

HR, Hazard ratio; MACEs, Major adverse cardiac events; CI, confidence interval; DM, Diabetes mellitus; AMI, Acute myocardial infarction; BMI, Body mass index; WBC,: White blood cell; TG, Triglyceride; LDL, Low-density lipoprotein; TC, Total cholesterol; CRP, C-reactive protein; EF, Ejection fraction; IR, Incomplete revascularization; IGF-1, Insulin like growth factor 1; IGFBP-2, Insulin like growth factor binding protein 2.

### MACEs prediction in ACS patients using receiver operator characteristics curves of circulating IGFBP-2 and EF

**Figure 3:**
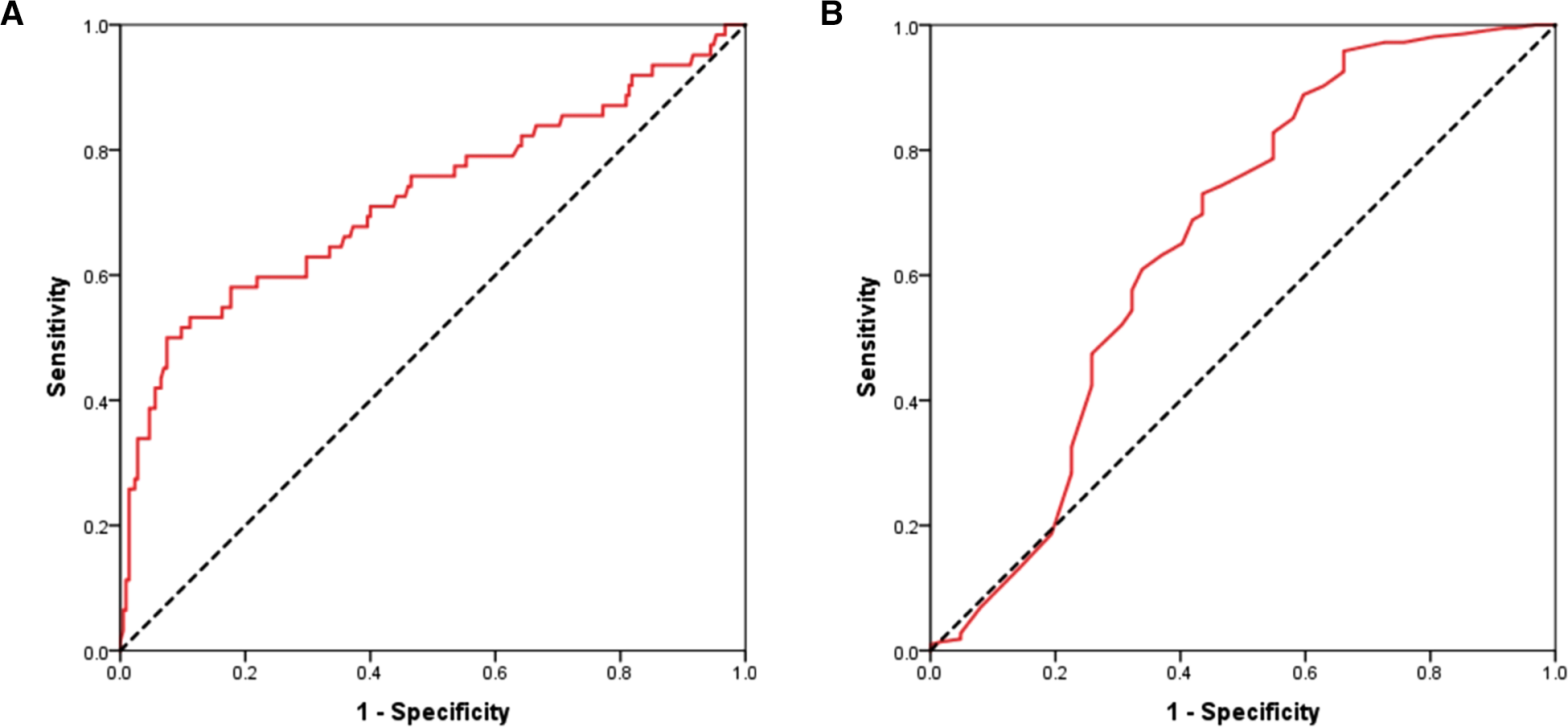
Receiver operating characteristic curves of circulating IGFBP-2 (**A**) and EF (**B**) for predicting MACEs in patients with ACS. IGFBP-2, Insulin like growth factor binding protein 2; EF, Ejection fraction; MACEs, Major adverse cardiovascular events; ACS, Acute coronary syndrome.

To evaluate the potential prognostic power of circulating IGFBP-2 and EF for MACEs prediction, ROC curves were generated. Analysis revealed that AUC values of plasma IGFBP-2 (Figure 3A) and EF (Figure 3B) for MACEs prediction in ACS patients were 0.722 (95% CI 0.640–0.804; *p* < 0.001) and 0.659 (95% CI 0.570–0.748; *p* < 0.001), respectively. No statistical differences in AUC values of IGFBP-2 and EF for MACEs prediction were found (Z = 1.02; *p* > 0.05).

## Discussion

We evaluated a cohort of 277 ACS patients and 42 healthy controls to investigate the relationship between IGF-1 and IGFBP-2 levels and prognosis for short-term outcomes. Our findings revealed that (1) circulating IGF-1 levels in healthy controls were higher than in NSTEMI or STEMI patients, and IGFBP-2 levels showed an opposite pattern; (2) EFS was poor in patients with high levels of IGFBP-2; and (3) high IGFBP-2 levels, but not low IGF-1 levels, independently predicted for MACEs in ACS patients who underwent CAG.

IGF-1 levels were previously found to be lower in acute MI patients compared to healthy controls (24, 25). To date, however, studies evaluating the influence of IGFBP-2 levels on cardiovascular disease remain scarce. In this study, we not only confirmed IGF-1 levels in healthy controls to have been significantly higher than those in acute MI patients, but also found IGFBP-2 levels to have been higher in acute MI patients than those in healthy controls. Levels of IGF-1 and IGFBP-2 were previously reported to associate with EF and potentially serve as biomarkers for HF (9, 26–28). In agreement with previous studies, we found that IGF-1 levels positively associated with EF, whereas IGFBP-2 negatively associated with EF. Although HF incidence was greater in high IGFBP-2 group patients, no differences between groups were found for IGF-1 levels. We noted that high IGFBP-2 group patients had lower triglyceride levels and BMI. Our findings are in agreement with prior literature that reported IGFBP-2 to be a marker of metabolic syndrome and inversely correlate with BMI and triglyceride levels (29–31).

No differences in incidences of MACEs or death were found between low and high IGF-1 group patients in this study. Although no difference in mortality was noted between high and low IGFBP-2 group patients, the incidence of MACEs in high IGFBP-2 group patients was found significantly higher as compared to low IGFBP-2 group patients. Moreover, after adjusting for age and other variables, we found that IGFBP-2 was an independent predictive factor for MACEs, while IGF-1 was not. Iswandi et al. (32) reported that IGF-1 was not an independent predictor of cardiovascular mortality or morbidity in ACS patients over a 5-year follow-up period. Furthermore, Wallander et al. (33) found that IGF-1 levels at hospital admission were not related to cardiovascular death over a three-year follow-up period in patients with type 2 diabetes who suffered acute MI. Although our findings were in agreement with most previously reported, a study by Bourron and et al. (34) reported that low IGF-1 levels not only associate with increased mortality risk but also with risk of any MACEs in acute MI patients over 2 years of follow-up. As various populations may yield disparate findings, future studies should confirm whether IGF-1 is predictive for adverse outcomes in diverse groups.

Increasing evidence has highlighted the cardioprotective effects of IGF-1 in the setting of cardiovascular disease. Numerous *in vivo* and *in vitro* studies reported that IGF-1 facilitates resistance to apoptosis in hypoxic conditions (8), stimulates fetal cardiac growth (7), increases production of circulating angiogenic cytokines (35) and exerts positive inotropic and antioxidant effects (36). Translational research has further revealed that treatment with IGF-1 significantly reduces left ventricular volume, attenuates left ventricular mass and improves stroke volume in STEMI patients (37), and improves EF in HF patients (38). However, Conover et al. (39) found that transgenic overexpression of pregnancy-associated plasma protein-A increases IGF-1 activity and results in accelerated atherosclerotic lesion development. Hirai et al. (40) reported that IGF-1 promotes atherosclerosis by affecting endothelial function and increasing aging in rabbits fed a cholesterol-rich diet. As such, the roles of IGF-1 in cardiovascular disease remain unclear and warrant further study.

Previously, IGFBP-2 was reported to inversely correlate with BMI (41). Indeed, studies reported that low IGFBP-2 independently associates with an increased risk of metabolic syndrome as well as elevated fasting glucose levels (30). Higher circulating IGFBP-2 concentrations were also longitudinally associated with lower type 2 diabetes risk (42, 43). Although IGFBP-2 is considered to protect against cardiovascular risk factors, high levels of IGFBP-2 were reported to associate with poor prognoses in several diseases. Prior studies reported that IGFBP-2 independently predicts for adverse clinical outcomes in patients with HF (16, 44), severe aortic stenosis (17), dilated cardiomyopathy (45) and PAH (46). To date, studies about IGFBP-2 in CVD is seldom, and the present study first uncovered that IGFBP-2 is an independent predictor of MACEs in ACS patients. Additionally, although no significant differences in prognosis power among IGFBP-2 and EF were noted, the AUC value of circulating IGFBP-2 was found to be greater than that of EF. Studies enrolling more eligible patients may suggest the prognostic power of IGFBP-2 was superior to EF in ACS patients. The seemingly paradoxical influences of IGFBP-2 on cardiovascular risk factors and pathological processes warrant detailed study.

The potential mechanism of IGFBP-2 and MACE is thought to be multifactorial. IGFBP-2 plays a crucial role in regulating mitogen-activated protein kinase (MAPK) pathway, which is a driver of atherosclerosis and involved in inflammatory signaling and oxidative stress (47, 48). Moreover, IGFBP-2 regulates the phosphatidylinositol 3-kinase (PI3K)/alpha serine/threonine-protein kinase (Akt) signaling pathway, which has a fundamental role in the pathological processes of atherosclerosis (49, 50). In addition, IGFBP-2 enhances the migration and proliferation of vascular smooth muscle cells (VSMC), this process is associated with the development of atherosclerosis (51). Further studies about the underlying mechanisms of IGFBP-2 in CVD are certainly warranted.

Our study, although well-designed, was not without limitations. First, the participants in this study were predominantly male, our sample size was relatively small and the follow-up period was short. As such, sex differences were not investigated, and the low incidence of mortality limited the hard endpoint analysis of the study. In addition, a lack of significant findings concerning IGF-1 and MACEs prediction may have occurred due to a type II statistical error. Second, the mechanisms behind the association between circulating levels of IGF-1 and IGFBP-2 and the incidence of MACE were not elucidated. Third, healthy controls were not well-matched with ACS patients for gender or age. Finally, blood samples were collected at admission and lacked data concerning serial fluctuations in IGF-1 and IGFBP-2 levels during follow-up. Therefore, multi-center studies with a greater sample size and animal studies about potential biological mechanisms of IGF-1 and IGFBP-2 are needed to confirm these results.

## Conclusion

Plasma IGFBP-2 levels were higher in patients who suffered acute MI compared to healthy controls. A high IGFBP-2 level, but not a low IGF-1 level, likely has clinical use as a prognostic biomarker for MACEs in patients with ACS. Although the underlying mechanisms for our findings remain unclear, we provide a foundation for further study of IGFBP-2 and improvements in clinical management of patients suffering ACS.

## Data Availability

The raw data supporting the conclusions of this article will be made available by the authors, without undue reservation.
